# Transactions of the Buffalo Obstetrical Society

**Published:** 1888-05

**Authors:** 


### Society Reports.

### *TRANSACTIONS OF THE BUFFALO OBSTETRICAL SOCIETY.*

(Stated Meeting, March 27, 1888.)

The President, Dr. R. L. Banta, in the chair.

Dr. Frank H. Potter read a paper, entitled "Notes on the Treatment of Tonsillitis in Children." [See page 455, this issue of the Journal.]

DISCUSSION.

Dr. Stockton said that his views of the constitutional treatment did not differ from those expressed in the paper; that diatheses, especially the rheumatic and gouty, should be borne in mind by the therapeutist; but that he was, however, unacquainted with the local treatment advocated by the essayist, and, consequently, could not speak in intelligent criticism thereof. He observed that the paper did not treat of or consider one distinct form of tonsillitis that had given him more trouble than any other, viz.: the asthenic or anæmic variety that occurs generally in delicate females, who have enfeebled circulations and were otherwise poorly nourished; these were, in his view, even more troublesome to manage than those cases depending upon the rheumatic or gouty diathesis. Guiaicum and biniodide of mercury had proved valuable remedies in his hands for such cases.

He, furthermore, regarded the disease as essentially different, when it invaded the deep structures of the gland, from that which only involved its lacunæ, i. e., whether it was a parenchymatous inflammation or a peritonsillitis, these being two distinct forms of the disease, pathologically speaking, as well as clinically.

The suppurative form of the malady was to be treated surgically; in the erythematous and follicular varieties, he gave one-fiftieth grain of the biniodide of mercury to the adult, proportionately to children, and had employed phytollacca decandra—dose, two to three drops frequently repeated—with benefit, though he confessed to the use of the latter remedy somewhat empirically.

Finally, he spoke of the value of hot water, either as a gargle or applied to the tonsils through the medium of pledgets of cotton, and, in a concluding compliment to the essayist, hoped he would meet with his usual good fortune in having his paper translated into four tongues, and read by the nations of the earth.

Dr. Hartwig had not expected much from the use of bicarb. of soda, and, therefore, had not employed it; but, from the statements of its efficacy by the reader, he should be induced to make a trial of it. He upheld the pathological views of the paper, but had grave doubts as to the cure of parenchymatous inflammation in two days by any method; certainly, such cases would go on to suppuration, no matter what was done. Inquiring what was thought of chipping off pieces of the tonsil during the progress of the disease, he proceeded to relate cases that he had seen cut short on the third day by the employment of this measure, but admitted that it was not usual to meet with purulent tonsillitis in a child under ten years.

Dr. Campbell confessed it was new to him that the secretions in these cases were acid, and inquired, "Is this an established fact? If so, what is the nature of the acid?" He regarded the exhibition of antifebrin as good treatment, stating that it was a more powerful nervous sedative than aconite; but believed some cases would recover in two or three days, in spite of any and all treatment, while it was equally true that others would run as many weeks, no matter what plan was pursued. He referred to the employment of the chloride of ammonia and phytolacca decandra, the latter used chiefly in the south, and the ammoniated tincture of guiaic, all useful remedies in this disease.

Dr. Briggs regarded the different types of the disease described by the members, and which were apparently so confusing, as explained, either by the different stages in which the cases were seen, or to the different forms that inflammatory action was well known to assume in different individuals. In a form of acute tonsillitis, ushered in with a chill and the other phenomena attendant thereupon, the treatment he had employed of late was morphia (grain, one-twentieth), and Fleming's tincture of aconite (one drop), administered every hour or two (adult dose—this proportion to children, according to age), and he had yet to see a case, taken early, where, under this plan, the inflammation had not subsided in from twelve to twenty hours, the patients sleeping quietly most of the time; whereas, under all other methods he had employed, they were restless, fretful and perturbed in spirit. As to local applications, he had no confidence in any excepting hot water, which he allowed if the patients were old enough to gargle.

In a chronic form of the disease, frequently encountered—he had seen two such cases that day—where the tonsils filled the whole faucial cavity, and yet, nevertheless, where the patients were about the house, could swallow moderately well, and speak, without muffled voice, with little or no apparent constitutional disturbance. What, he asked, was to be done in such cases? They were, to his mind, most difficult cases to manage. He had seen scarification recommended and even practiced, but was yet in doubt as to its usefulness. He had no experience with the alkaline method proposed by the essayist in the acute tonsillitis of children; but in anæmic cases, and many of the recurrent types were so, he approved of the general after-treatment recommended.

Dr. Van Peyma's attention had been called by the essayist to this method of treatment some time ago, and he had tried it in a few cases. One adult, the wife of a butcher, said she "liked the powders," and did well under them; another case, a child, recovered promptly; but a third case did not do as well, though this was not a typical case, as the disease, though involving the tonsil, was not limited to it. He was always much interested in any discussion involving the various kinds of inflammation, and had tried, in his studies, to define the boundaries of the inflammatory processes; but had finally come to the conclusion that, very much like the history of snakes in Ireland, there were no boundaries, and this fact, he thought, was not emphasized as it should be.

He believed the various kinds of inflammation described in general were more the differences of degree, and of the varying histological characters of the tissues involved, than of real differences of the disease itself. In regard to the employment of phytolacca decandra in tonsillitis, he desired to call the attention of the society to the somewhat analogous use of the drug, recommended in a paper on diseases of the puerperal breast he had presented here some two years ago.

Dr. Thornton commented upon the paper as being short and to the point, though it omitted to treat of cases we so often met, where the inflammation had already gone on to suppuration when first seen by the physician. He had been in the habit of using the chlorate of potash and iron combination in follicular tonsillitis, and had also employed aconite with satisfaction, but would now use antifebrin instead of the latter, as recommended by the essayist. He was somewhat at a loss to account for the fact that an acid mixture and the alkaline treatment should both apparently relieve this disease with tolerable certainty.

Dr. Howell referred to the point made by Dr. Campbell, as to the chemical composition of the acid found in the secretions, and inquired: "Are these secretions markedly acid in all cases?" He confessed some surprise at the efficacy of the bicarb. soda, which appears, according to the essayist, to have aborted the attacks. His own custom had been to give aconite in the early stages of the disease, and to also employ an antiseptic and deodorant gargle, by which plan he aimed to reduce pyrexia, remove offensive secretions, and destroy germs. In the later stages, he has employed tincture of belladonna in one-drop doses every two hours with success. His experience with guiaicum was limited to a single case, and as the employment of this drug caused the patient to seek advice at other hands, he had never had the courage to prescribe it again.

Certain subjects, as well known, were prone to recurrent attacks, and, as an apparent consequence, had habitually enlarged tonsils. Whether the fibrosis in such cases contributed to the subsequent attacks by impairing the circulation, or whether these were due to diatheses or dyscrasias, he could not say; but of one thing he was very sure, that resection of the tonsils was good treatment in such conditions; and another point that had become settled in his mind was, that exsection was even proper in their acute stages. Hemorrhage need not be feared as a consequence of the operation, as it could be readily controlled by diluted vinegar.

Dr. Potter, in closing the discussion, said the remarks to-night had reminded him of the literature of the subject; almost every author had his favorite method of treatment which he believed better than all others. He thought this diversity of opinion due to the fact that the various degrees of inflammation of the tonsil had been classified and described as so many distinct diseases; his own belief being that after an inflammation was started in that organ, its expression was determined by the kind of soil upon which it grew. Dr. Van Peyma had made a most wise observation when he said the limits of an inflammation could not be sharply defined. It was the non-recognition of this fact that made opinions so diverse and treatment so involved. The indications were simply to treat the diathesis, where such a constitutional condition was recognized, to allay the fever, and to relieve the local distress. The shortest way to this end was the most rational treatment. The degree of the disturbance determined the necessity for the more or less energetic application of the method adopted. The various suggestions made by the members were often of benefit, but they were adapted to special conditions, and failed to recognize the unity of the inflammations of the tonsil. The method proposed in the paper was simple and effective, and particularly adapted to children, which, he begged the society to remember, was his chief purpose in bringing the subject before it.

In regard to the reaction of the secretions of the mouth during these attacks, he would say that, in his cases, they were acid. This was not remarkable, for the acid secretion occurs sometimes in health, as after severe exercise, etc., and it was known that the secretions from the nose in acute rhinitis were acid.

